# First identification of *Tyrophagus curvipenis* (Acari: Acaridae) and pathogen detection in *Apis mellifera* colonies in the Republic of Korea

**DOI:** 10.1038/s41598-023-36695-z

**Published:** 2023-06-10

**Authors:** Thi-Thu Nguyen, Mi-Sun Yoo, A-Tai Truong, Jong Ho Lee, So Youn Youn, Se-Ji Lee, Dong-Ho Kim, Soon-Seek Yoon, Yun Sang Cho

**Affiliations:** 1 Laboratory of Parasitic and Honey Bee Diseases, Bacterial Disease Division, Department of Animal and Plant Health Research, Animal and Plant Quarantine Agency, Center for Honey Bee Disease Control, 39660 Gimcheon, Republic of Korea; 2 Plant Pest Control Division, Department of Plant Quarantine, Animal and Plant Quarantine Agency, Gimcheon, 39660 Republic of Korea; 3 Faculty of Biotechnology, Thai Nguyen University of Sciences, Thai Nguyen, Vietnam

**Keywords:** Parasitology, Pathogens

## Abstract

Mites of the genus *Tyrophagus* (Acari: Acaridae) are among the most widely distributed mites. The species in this genus cause damage to stored products and crops, and pose a threat to human health. However, the influence of *Tyrophagus* spp. in apiculture remains unknown. In 2022, a study focusing on the identification of *Tyrophagus* species within five apiaries was conducted in Chungcheongnam Province, Republic of Korea. Its specific objective was to investigate the presence of *Tyrophagus* mites in response to the reported high mortality of honey bee colonies in this area. Morphological identification and phylogenetic analysis using the mitochondrial gene cytochrome-c oxidase subunit 1 (*CO1*) confirmed for the first time the presence of the mite species *Tyrophagus curvipenis* in a honey bee colony in the Republic of Korea. Two honey bee pathogens were detected in the mite, a viral pathogen (deformed wing virus, DWV) and a protozoal pathogen (*Trypanosoma* spp.). The presence of the two honey bee pathogens in the mite suggests that this mite could contribute to the spread of related honey bee diseases. However, the direct influence of the mite *T. curvipenis* on honey bee health remains unknown and should be further investigated.

## Introduction

Honey bees (*Apis mellifera*) are essential pollinators of crops and wild plants and play an indispensable role in human life by providing important products such as honey, royal jelly, and propolis. However, the loss of honey bee colonies due to climate change and the spread of pathogens has occurred worldwide^1–3^. One of the leading causes of honey bee deaths are mites such as *Varroa* spp. and *Tropilaelaps* spp., which feed on honey bees and are vectors for disease transmission in honey bees^4–7^. Vector-mediated transmission significantly increases the spread of pathogens, contributing to the collapse of the infected bee colony. Recent increase in the rate of honey bee colony loss has become a great concern for beekeepers and researchers; the rate of colony loss has been as high as 36.5% in many countries^8^, and a loss of approximately 37.7% was reported in 2018–2019 in the United States^9^. In the Republic of Korea (ROK), the value of honey bee pollination has been estimated at $5.9 billion, corresponding to approximately 50% of the economic value of fruit and vegetable production^10^; however, the loss of about 18% of honey bee colonies in the country has been contributed to unknown reasons^11^. Therefore, it is crucial to identify the factors that adversely affect beekeeping in the country.

Mites of the genus *Tyrophagus* are distributed worldwide in a range of habitats, including natural and anthropogenic, as well as plant and animal hosts^12–14^. Several species of *Tyrophagus* damage economic crops such as greenhouse vegetables and ornamental flowers^15,16^. *Tyrophagus* belongs to the superorder Acariforms, family Acaridae, and it includes approximately 35 species recorded globally^12,17^. *Tyrophagus curvipenis* (Acari: Acaridea) has been reported in a variety of animal hosts in Costa Rica and Russia, ^18^ and in several plants in New Zealand, France, Australia, and Portugal^12^. Fain and Fauvel first reported *T. curvipenis* mites isolated from wooden structures of a greenhouse in Portugal, and they^19^ recorded that mites occasionally inhabit the flowers of orchids and may feed on their pollen^19^. Several species of *Tyrophagus* (such as *T. putrescentiae, T. curvipenis, T. tropicus, T. debrivorus, T. similis, T. longgior, T. mixtus, T. perniciosus, T. vanheurni*, and *T. savasi*) have been associated with bees^20^. *Tyrophagus putrescentiae* (Acari: Acaridae) was detected in dead honey bee samples^21^, in the body of bumblebees^22^, and in honey bee hives in Brazil. This suggests the potential of the mites to harm human health upon consumption of the products from contaminated honey bees^23^. No study reports the presence of the remaining nine species of *Tyrophagus* in honey bees. Acting as potential carriers of pathogens or vectors for pathogen infections, these mites can affect humans indirectly^24–26^; therefore, the role of *T. curvipenis* and other species of mites infecting honey bees need further studies and evaluation.

Identification of *Tyrophagus* mites has been traditionally based on morphology^12,14,27^. However, this method is laborious due to the small size of *Tyrophagus* mites, requires good understanding of morphological traits, and it is time-consuming. The molecular method based on the mitochondrial gene cytochrome-c oxidase subunit 1 (*CO1*) and the nuclear ribosomal internal transcribed spacer 2 (ITS2) was suggested as an alternative tool for species identification of microscopic mites^28^.

Screening for the factors responsible for the honey bee colony loss was carried out in the dead colonies collected from the regions with high colony collapse reported in winter. Here, mites were detected and collected from dead honey bee colonies in the ROK in 2022. The *CO1* and ITS2 sequences were analyzed to identify the mite species. In addition, honey bee pathogens carried by the mite were also identified.

## Results

### Morphological identification of mites

**Figure 1:**
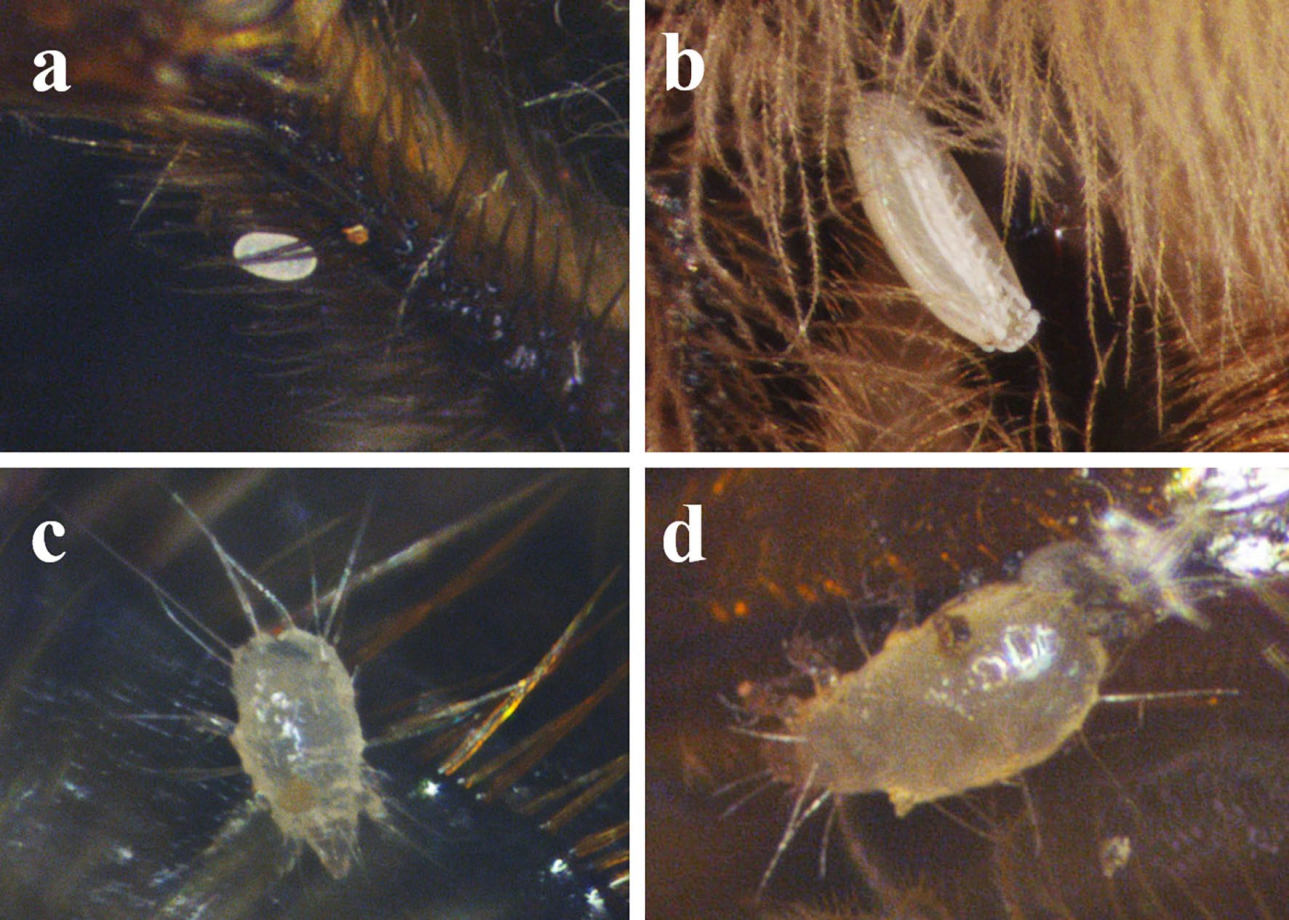
Different stages of *Tyrophagus curvipenis* detected in honey bees (*Apis mellifera*)*.* Identity of acaroid mites was determined by analyzing morphological characteristics using a phase contrast microscope. Different living stages of the mite are presented: egg (**a**), larva (**b**), nymph (**c**), and adult (**d**).

**Figure 2:**
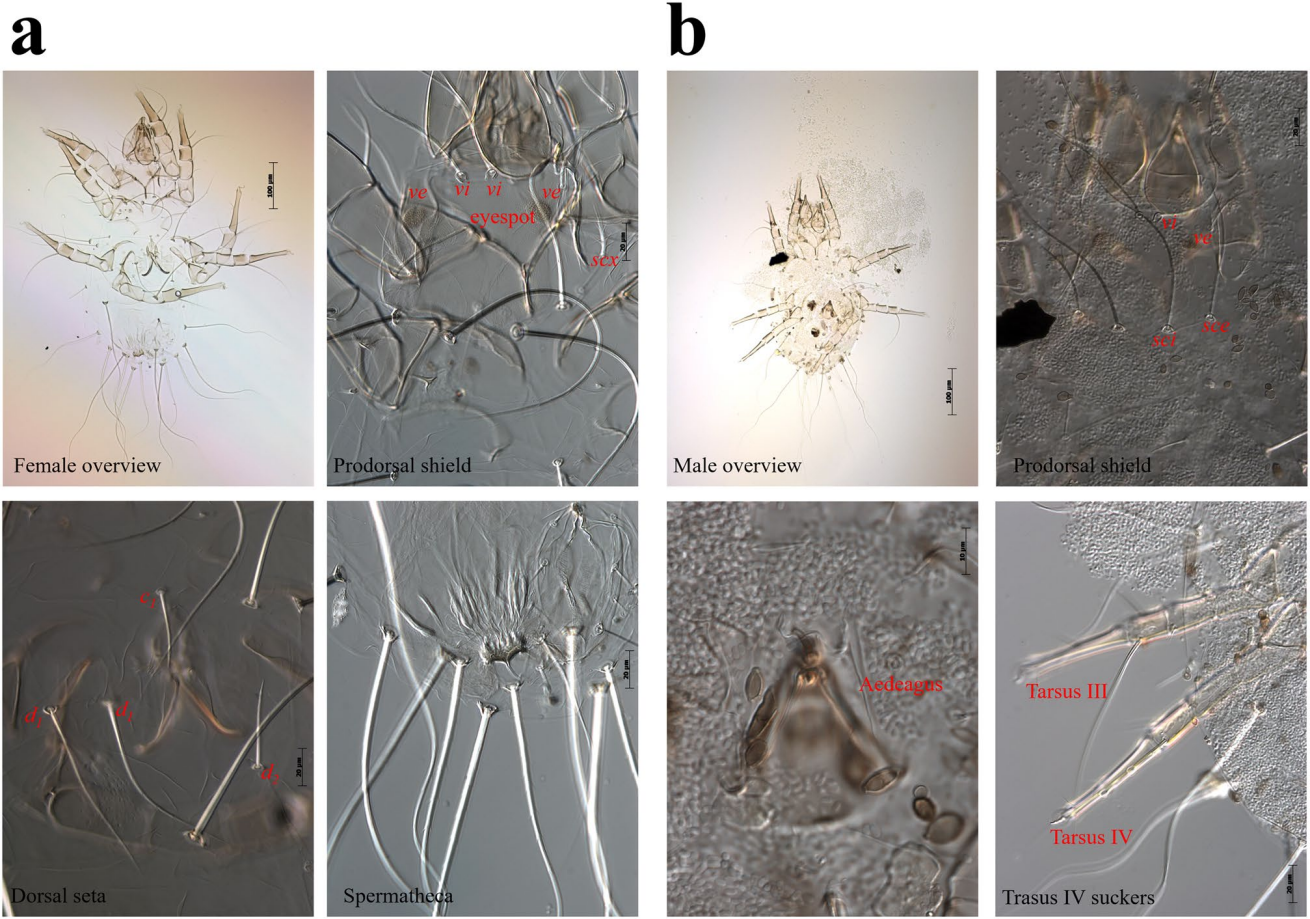
Morphological characterization of *Tyrophagus curvipenis*. Identification of *Tyrophagus* mites was based on the position of setae *ve*, the shape of setae *scx.* (**a**) Female, the comparison of the size of c1, d1, and d2 setae to the distance between these setae and the posterior setae, and the shape of female spermatheca, (**b**) Male, S-shaped aedeagus and two suckers evenly distributed on tarsus IV.

Microscopic examination of the honey bee samples revealed the presence of *T. curvipenis* mites within their bodies, with an infestation rate of 100%. The mites isolated from the honey bee colony were associated with different life stages of the collected honey bee samples, namely the egg, larva, nymph, and adult stages (Fig. 1a–d). The *Tyrophagus* genus exhibits a distinct characteristic in adult mites, with their idiosoma appearing whitish to semitransparent (Fig. 1d). Identification of the mite species *T. curvipenis* Fain & Fauvel (Acari: Astigmata: Acaridae) was based on female and male characteristics. The female mites were small, pale, idiosoma 415–451 µm (*n* = 2) long, 218–225 µm wide; chelicera 82–86 µm; prodorsal shield nearly pentagonal, eye spots prominent, 83–84 µm long, 86–84 µm wide; supracoxal seta (scx, 31–36 µm) slender, with four moderate or short pectinations; Grandjean's organ finger-like, its basal lobe with two spiniform teeth; hysterosomal setae c1, d1, and d2 relatively shorter than others; c1 (34–42 µm) a bit shorter than d2 (35–47 µm), d1 (117–109 µm) approximately 3.4 × the length of c1 and 2.6 × the length of d2; spermathecal duct narrow, base of spermathecal sac r-shaped (Fig. 2a); coxal plate II broadly triangular, extending distally beyond the apex of apodeme II, with 1/3 of posterior margin slightly concave; tarsus I ω1 slender, cylindrical and slightly widened at apex, tarsus II ω slender, almost cylindrical; setae ω and r of tarsus IV setiform (Supplementary Fig. S1).

The male was smaller than the female, idiosoma 319 µm (*n* = 1) long, 196 µm wide. Chelicerae 71 µm; eyespots, coxal plates I and II, and solenidia I ω1 and II ω as in female; aedeagus curved into S-shape; two suckers evenly distributed on tarsus IV (Fig. 2b).

### Genetic identification of mite species

**Figure 3:**
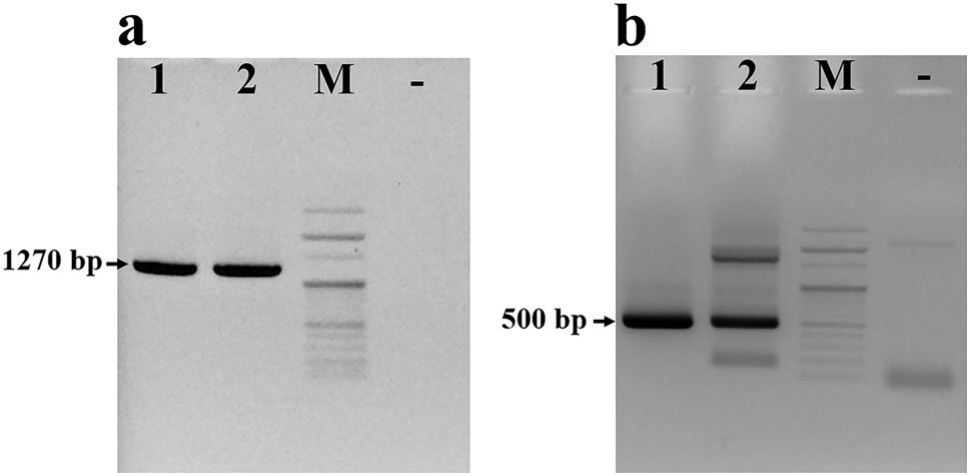
Amplification of *CO1* and ITS2 regions from mites isolated from honey bees. (**a**) PCR product of *CO1*, (**b**) PCR product of ITS2. PCR products were confirmed on 1% agarose gel. Lane M is 100 bp DNA ladder; lanes 1 and 2 are PCR products of the egg and adult samples, respectively. lane “-” is negative control without a DNA template. Amplicon size from sample DNA is shown in base pair (bp).

The family to which this species belongs can be determined based on the morphological characteristics observed under the microscope. Although morphological traits are highly useful for initial identification, accurately distinguishing the exact species can be challenging. To confirm the identification, species-specific primer pairs targeting the *CO1* gene and the ITS2 gene regions of *Tyrophagus* mites were designed. We successfully optimized the PCR reaction conditions for amplifying the *CO1* and ITS2 genes. The sequences were then determined through sequence analysis. Amplification of the *CO1* and ITS2 regions from adult mite and egg samples produced bands with expected length of 379 and 500 bp, respectively (Fig. 3a). Sequences of the amplified regions were 100% similar between the adult and egg samples. The search of the obtained *CO1* sequences against the GenBank database (blast.ncbi.nlm.nih.gov) revealed 100% sequence identity with *T. curvipenis* originating from Costa Rica (NCBI accession No.: KY986270) and 86.64% similarity with *T. putrescentiae* (NCBI accession No.: MH262448). The IST2 sequences of *T. curvipenis* are not available in the NCBI database, and the search of the sequences isolated from the samples against the GenBank database indicated the highest similarity of 91.78% to *T. putrescentiae* in China (NCBI accession No.: GQ205623.1)*.* In addition, non-specific band (around 1.7 kb long) was seen in the ITS2 amplification from adult sample (Fig. 3b). The unexpected band was extracted for sequencing. However, the result was unidentified due to the poor sequencing result.

**Figure 4:**
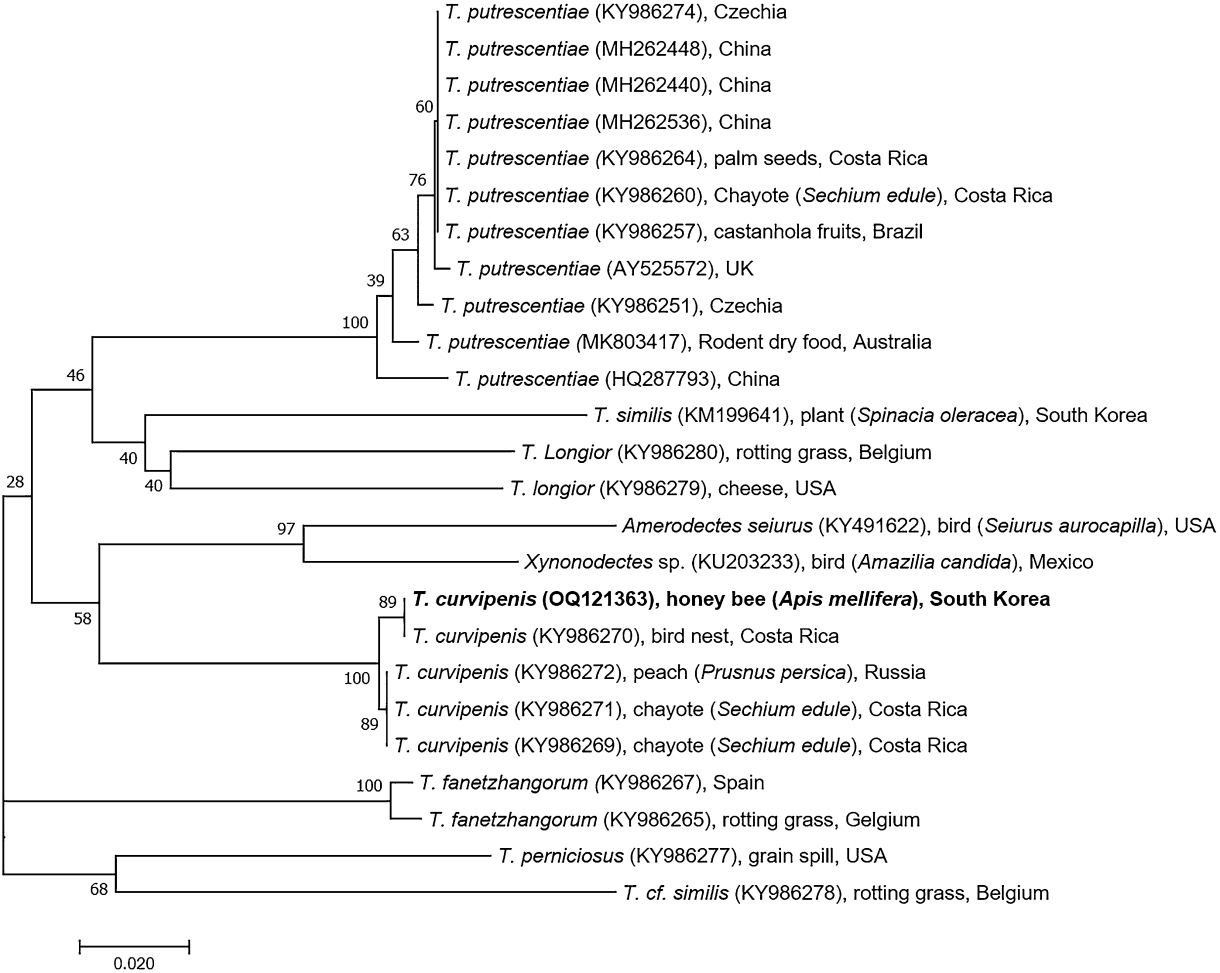
Phylogenetic neighbor-joining tree based on sequences of *CO1* gene. Species name, NCBI accession numbers, and origin of the accession are given for each sequence. The mite identified in honey bee samples in this study is in bold.

Phylogenetic analysis based on the *CO1* gene sequences placed the collected mite sister with *T. curvipenis* isolate AD2025 collected from a bird nest in Costa Rica and both were in the same clade with *T. curvipenis* isolated from plants (peach and chayote) in Russia and Costa Rica (Fig. 4).

### Detection of honey bee pathogens in mites

**Figure 5:**
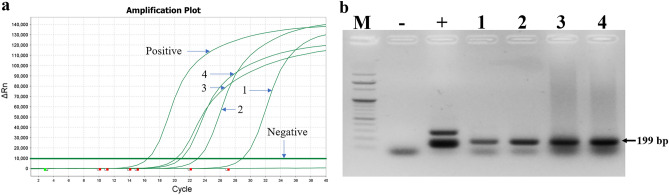
Detection of deformed wing virus (DWV) from honey bee and mite samples. Positive detection of DWV was identified in amplification curves of RT-qPCR (**a**). Results were confirmed by visualizing the expected band (199 bp long) on agarose gel (**b**). Lane M is 100 bp DNA marker. Lanes 1 and 2 are bands amplified from egg and adult samples of *Tyrophagus curvipenis*, respectively*.* Lanes 3 and 4 indicate the results from honey bee samples collected from two colonies. Lane “−” is negative control without DNA template, and lane ‘+’is positive control using recombinant DNA of DWV.

**Figure 6:**
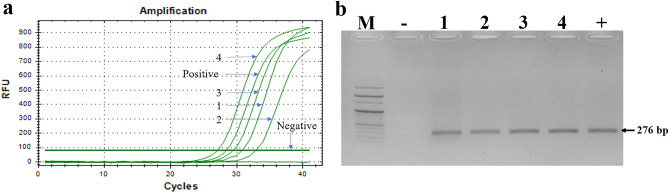
Detection of *Trypanosoma* from honey bee and mite samples. Positive detection of *Trypanosoma* in real-time PCR (**a**) was confirmed with the expected band (276 bp) on electrophoresis agarose gel (**b**). Lanes 1 and 2 indicate the results of detection from egg and adult samples of *T. curvipenis*, respectively. Lanes 3 and 4 are the result of *Trypanosoma* detection from honey bee samples collected from two different colonies. Line ‘−’ is negative control without DNA template, and line ‘+’ is positive control using *Trypanosoma* recombinant DNA.

Pathogen detection from 15 honey bee samples showed that the bee samples were infected with DWV, BQCV, and *Trypanosoma* (Supplementary Table S1). Therefore, to identify the possibility of the honey bee pathogen infection in the mites, we conducted pathogen detection using total nucleic acids extracted from the mites. Two honey bee pathogens (DWV and *Trypanosoma*) were detected in eggs and adult samples of the mite. Additionally, the two pathogens were present in the honey bee samples from the same colonies where the mites were collected. The identity of the pathogens was confirmed using reverse transcription real-time PCR (RT-qPCR), which produced bands of 199 bp and 276 bp for DWV and *Trypanosoma*, respectively (Figs. 5 and 6).

## Discussion

In this study, *Tyrophagus* mites were detected and identified in the colonies of five apiaries in Gongju, Chungcheongnam Province, Republic of Korea. This is the first report of *T. curvipenis* in *A. mellifera* colonies in the country*.* Morphological identification and genetic identification using *CO1* gene revealed that the mite belongs to the species *T. curvipenis*, and phylogenetic analysis of the obtained sequence indicated a close relationship between the *T. curvipenis* from the Republic of Korea and the isolate from a bird’s nest in Costa Rica (Fig. 4). In addition, the species phylogeny of *Tyrophagus* based on nucleotide similarity in the *CO1* gene region showed that the *T. curvipenis* detected in honey bee in this study was in the same cluster as that identified in plants such as peach and chayote from Russia and Costa Rica. The result suggests that the source of *T. curvipenis* identified in this study could be a flower visited by the honeybee for pollen and nectar collection. Four of the 35 identified species in the genus *Tyrophagus* have been reported to date in the Republic of Korea: *T. putrescentiae*, *T. similis*, *T. neiswanderi*, and *T. longior*^29–31^. *Tyrophagus curvipenis* reported here is thus a newly recorded species in the ROK. Similar to other species, *T. curvipenis* has been found in honey bee hives in New Zealand^32^. It was reported that *T. putrescentiae* in Korea (Wanju-gun, Jeonnam) inhabits beehives and feeds on debris and pollen^33^. However, the relationship between honey bee health and the presence of *T. curvipenis* mite in the hive remains unknown.

The molecular method was successfully used to identify *Tyrophagus* species. The result of the molecular method based on the *CO1* gene sequence was consistent with the identification based on morphological characteristics. However, due to the limited availability of *Tyrophagus* sequences in the NCBI database, species identification of the *Tyrophagus* mite could not be confirmed using the IST2 region. In addition, the IST2 primer pair amplified a non-specific band using the total DNA extracted from adult mites (Fig. 3b). The *CO1* gene, owing to a larger deposited sequence database, is potentially more beneficial for species identification of *Tyrophagus* mites.

Infestation of honey bees with hemolymph-feeding mites such as *Varroa* and *Tropilaelaps* increased colony loss over the winter season and transmission of honey bee diseases^4,6,7,34–36^. The viral, bacterial, and fungal honey bee pathogens harbored and transmitted by *Varroa* and *Tropilaelaps* mites have been demonstrated^36,37^. The *T. curvipenis* mite detected in this study was positive for a honey bee viral pathogen (DWV) and protozoal pathogen (*Trypanosoma*). The infection with these pathogens weakens the colony and increases the winter mortality of honey bees^38–40^. Additionally, these pathogens were detected in the honey bee samples where the mites were collected, suggesting a high possibility of mite transmitting such pathogens in the hives. Furthermore, *Tyrophagus* mites, commonly known as storage mites, feed on mold found in food^41,42^. However, the food sources of *T. curvipenis* in honey bee hives remain unknown. Therefore, understanding the influence of *T. curvipenis* mite on honey bees would help to develop appropriate methods for mite prevention and control. In addition, it is necessary to minimize the potential effects of the mites on humans who consume the honey bee products collected from infested colonies, because some *Tyrophagus* mites were identified as allergens in animals and humans^43,44^. With an observed infestation rate of 100% and the potential to serve as intermediate disease vectors within honey bee colonies in the five apiaries studied, *Tyrophagus* mites potentially play an important role in the observed colony losses. The findings from this study have the potential to contribute to the development of targeted management strategies aimed at minimizing the impact of *Tyrophagus* mite infestation on honey bee health and improving overall colony survival rates. By understanding the significance of *Tyrophagus* mites as potential contributors to colony decline, proactive measures can be implemented to mitigate their negative effects and safeguard the well-being of honey bee populations.

This is the first record of *T. curvipenis* mites in honey bee colonies in the Republic of Korea, and this study demonstrated the presence of honey bee pathogens (DWV and *Trypanosoma*) in mites. The result suggests that the mite could have an important role in spreading the honey bee pathogens. However, whether *T. curvipenis* mite contributed to the death of honey bee colonies has not been confirmed in this study; therefore, further study is necessary to understand the influence of this enemy and biological vector of honey bee pathogens in apiculture, and to reveal the potential risk of mites to humans consuming the products collected from infested honey bee colonies.

## Materials and methods

### Mite collection and morphological identification

Honey bee samples were collected from dead colonies in apiaries in Gongju-si, Chungcheongnam Province, ROK, in November 2022. A total of 45 honey bee samples were collected from 15 colonies in five different apiaries for mite examination. The presence of mites was confirmed under a dissecting microscope. The mites were collected from the body surface and hairs of honey bees. Mites were mounted on slides with Hoyer's medium. The specimens were identified and measured according to Fan and Zhang^12^. All the measurements used herein are in micrometers. After species identification, the mites were used for genetic analysis and pathogen detection.

### Genomic DNA extraction and total nucleic acid extraction

Honey bee samples and collected mites were washed three times using UltraPure™ distilled water (Invitrogen, USA) and used for total nucleic acid extraction. The Maxwell RSC viral total nucleic acid purification Kit (Promega, USA) was used for nucleic acid extraction according to the manufacturer’s instructions. The extracted nucleic acids were used for the detection of honey bee pathogens. Extraction of the mite genomic DNA (gDNA) was done using a QIAamp DNA Mini Kit (Qiagen, UK) according to the manufacturer’s instructions with some modifications^45^. Ten adult mites or four mite eggs collected from each honey bee colony were pooled and placed in a 1.5 mL tube containing 200 µL ATL buffer and 2.381 mm steel beads (Hanam, ROK). After homogenizing at 5000 rpm for 15 s, 20 µL proteinase K (50 µg/mL) was added, and the mixture was incubated at 56 °C for 1 h. Exactly 200 µL of lysis buffer was added to the solution, and the solution was incubated for 10 min at 70 °C. Next, 200 µL of 98% ethanol was added, and the mixture was mixed by vortexing. The solution was transferred to the AIAamp mini spin column and centrifuged at 12,000×*g* for 1 min. After washing the spin column twice with the washing buffer, the genomic DNA of the mites was eluted into a new tube using the elution buffer and centrifuged at 12,000×*g* for 2 min. The isolated DNA was used for PCR amplification of the *CO1* and ITS2 regions.

### Detection of honey bee pathogens

The mite and honey bee samples from each colony were tested for the presence of honey bee pathogens using the total nucleic acid of honey bee and mite samples. The honey bee pathogens targeted for detection included American foulbrood (AFB), European foulbrood (EFB), *Ascosphaera apis* (ASCO), *Aspergillus flavus* (ASP), *Nosema*, *Acarapis woodi* (ACAR), *Apocephalus borealis* (PHORID), Sacbrood virus (SBV), DWV, Black queen cell virus (BQCV), Chronic bee paralysis virus (CBPV) Kashmir bee virus (KBV), Acute bee paralysis virus (ABPV), Israeli acute paralysis virus (IAPV), *Apis mellifera* filamentous virus (AmFV), clades of Lake Sinai virus (LSV1, LSV2, LSV3, LSV4), *Trypanosoma*, *Varroa destructor virus*-1 (VDV-1; DWV-B), and recombinant Deformed wing virus-*Varroa destructor virus*-1 (DWV-VDV-1). Detection of pathogens was conducted in honey bee samples using RT-qPCR Kits (LiliF ABPV/KBV/IAPV/CBPV Real-time RT-PCR Kit, LifiF SBV/KSBV/DWV/BQCV Real-time RT-PCR Kit [iNtRON Biotechnology, Inc., ROK], Pobgen bee pathogen detection Kit [Postbio, ROK], and *iTaq* Universal SYBR green one-step Kit [Bio-Rad, USA]). Primers used for the detection of honey bee pathogens are presented in Supplementary Tables S2 and S3. The pathogens detected in honey bee samples were targeted for detection in mite samples using the PCR kits.

### PCR amplification and sequencing of mite DNA

The *CO1* and ITS2 regions of mites were amplified using universal primer sets *CO1*-forward (5ʹ-GTTTTGGGATATCTCTCATAC-3ʹ) and *CO1*-reverse (5ʹ-GAGCAACAACATAATAAGTATC-3ʹ)^46^; and ITS2-forward (5ʹ-CGACTTTCGAACGCATATTGC-3ʹ) and ITS2-reverse (5ʹ-GCTTAAATTCAGGGGGTAATCTCG-3ʹ)^47^. Each reaction mixture (20 µL) was composed of 20 ng of gDNA template, 1 µL of each primer (10 pmol), AccuPower® PCR preMix and master Mix (Bioneer, Korea), and ddH_2_O (to make up to 20 µL). The PCR thermal cycler protocol was optimized as follows: 94 °C (5 min); 5 cycles of 94 °C (20 s), 52 °C (30 s), and 68 °C (30 s); followed by 5 cycles of 94 °C (20 s), 50 °C (30 s), and 68 °C (30 s); 30 cycles of 94 °C (20 s), 48 °C (30 s), 68 °C (30 s); and the final 30 cycles of 94 °C (20 s), 46 °C (30 s), 68 °C (30 s); and the final extension step at 68 °C (5 min). After confirming the bands of *CO1* (379 bp) and ITS2 (500 bp) using agarose gel electrophoresis, the bands were extracted and purified using a QIAquick gel extraction Kit (Qiagen). The purified PCR products were sequenced by Cosmogenetech Co, Ltd. (ROK).

### Phylogenetic analysis

Identified nucleotide sequences from different adult and egg samples were subjected to BLASTn search against the NCBI nucleotide database. The nucleotide sequences were aligned using BioEdit and Cluster W software^48,49^. The phylogenetic tree based on the *CO1* sequence was created using the neighbor-joining method with 1000 bootstrap replications^50^ in MEGA7 software^51^.

## Supplementary Information

Supplementary Information.

## Data Availability

All data generated or analyzed during the current study are available in the National Center for Biotechnology Information (NCBI) repository, accession number: OQ121480 (ITS2) and OQ121363 (*CO1*).
