# Supplementary material for: First identification of Tyrophagus curvipenis (Acari: Acaridae) and pathogen detection in Apis mellifera colonies in the Republic of Korea
Source: Sci Rep. 2023 Jun 10;13:9469. doi: 10.1038/s41598-023-36695-z (PMC10257717; doi:10.1038/s41598-023-36695-z)
Supplement: Supplementary file 1 — Supplementary Information. [file 41598_2023_36695_MOESM1_ESM.docx]

Supplementary material

First identification of *Tyrophagus curvipenis* (Acari: Acaridae) and pathogen detection in *Apis mellifera* colonies in the Republic of Korea

Thi-Thu Nguyen, Mi-Sun Yoo, A-Tai Truong, Jong Ho Lee, So-Youn Youn, Se-Ji Lee, Dong-ho Kim, Soon-Seek Yoon, Yun Sang Cho*

*** Correspondence:** Yun Sang Cho: choys@korea.kr

**Table S1. Detection of viral pathogens in honey bee samples**

| **Honey bee Samples** | **Pathogens** | | | | | | | | | | | | | | | | | | | | | | | |
| --- | --- | --- | --- | --- | --- | --- | --- | --- | --- | --- | --- | --- | --- | --- | --- | --- | --- | --- | --- | --- | --- | --- | --- | --- |
|  | **AFB** | **EFB** | **ASCO** | **ASP** | ***Nosema*** | **ACAR** | **PHORID** | **SBV** | **DWV** | **BQCV** | **KBV** | **ABPV** | **CBPV** | **IAPV** | **LSV1** | **LSV2** | **LSV3** | **LSV4** | **AmFV** | **DWV-B** | **VDV-DWV** | ***Trypanosome*** | ***T. curipenis*** |  |
| **1** | - | - | - | - | - | - | - | - | 26.23 | 37.63 | - | - | - | - | - | - | - | - | - | - | - | 25.64 | + |  |
| **2** | - | - | - | - | - | - | - | - | 22.13 | 34.96 | - | - | - | - | - | - | - | - | - | - | - | 25.89 | + |  |
| **3** | - | - | - | - | - | - | - | - | 33.89 | 36.95 | - | - | - | - | - | - | - | - | - | - | - | 29.57 | + |  |
| **4** | - | - | - | - | - | - | - | - | 33.25 | 36.89 | - | - | - | - | - | - | - | - | - | - | - | 22.38 | + |  |
| **5** | - | - | - | - | - | - | - | - | 24.00 | 37.19 | - | - | - | - | - | - | - | - | - | - | - | 33.88 | + |  |
| **6** | - | - | - | - | - | - | - | - | 25.24 | 35.66 | - | - | - | - | - | - | - | - | - | - | - | 22.57 | + |  |
| **7** | - | - | - | - | - | - | - | - | 29.09 | 35.73 | - | - | - | - | - | - | - | - | - | - | - | 31.14 | + |  |
| **8** | - | - | - | - | - | - | - | - | 32.39 | 37.87 | - | - | - | - | - | - | - | - | - | - | - | 25.02 | + |  |
| **9** | - | - | - | - | - | - | - | - | 21.64 | 36.4 | - | - | - | - | - | - | - | - | - | - | - | 18.1 | + |  |
| **10** | - | - | - | - | - | - | - | - | 28.16 | 36.99 | - | - | - | - | - | - | - | - | - | - | - | 19.07 | + |  |
| **11** | - | - | - | - | - | - | - | - | 27.85 | 37.9 | - | - | - | - | - | - | - | - | - | - | - | 31.07 | + |  |
| **12** | - | - | - | - | - | - | - | - | 22.79 | 36.17 | - | - | - | - | - | - | - | - | - | - | - | 31.77 | + |  |
| **13** | - | - | - | - | - | - | - | - | 30.95 | 37.07 | - | - | - | - | - | - | - | - | - | - | - | 30.11 | + |  |
| **14** | - | - | - | - | - | - | - | - | 24.65 | 37.96 | - | - | - | - | - | - | - | - | - | - | - | 35.01 | + |  |
| **15** | - | - | - | - | - | - | - | - | 30.64 | 36.92 | - | - | - | - | - | - | - | - | - | - | - | 30.1 | + |  |

**Table S2. Primers and probes used for detection of viral pathogens**

| No. | Target | Primer | Sequence (5ꞌ→3ꞌ) | Amplicon size (bp) | Reference |
| --- | --- | --- | --- | --- | --- |
| 1 | SBV | SBV-F | AGAAGACATTTGATACAGTGGACTC | 131 bp | ^1^ |
|  |  | SBV-R | GGAATTCCAGATTCTTCGTCCAC |  |  |
|  |  | Probe | FAM–GATTTGTTTAATGGTTGGGTTTCTGGTA–BHQ-1 |  |  |
| 2 | DWV | DWV-F | TTCAACTCGGCTTTCTACGG | 170 |  |
|  |  | DWV-R | GTGTCTTTTTCTCTTTCTGACACC |  |  |
|  |  | Probe | ROX- ATGTCAACATTGGTATGCTCCGTTGAC- BHQ-2 |  |  |
| 3 | BQCV | BQCV-F | CCTTTGGCAATAGAACAAATACC | 143 |  |
|  |  | BQCV-R | GTGGCTATATCGAGATTATTCCG |  |  |
|  |  | Probe | Cy5- AGTCGCAGAGTTCCAAATACCGTACTATG- BHQ-3 |  |  |
| 4 | ABPV | ABPV-F | TGCCCTATTTAGGGTGAGGAG | 239 |  |
|  |  | ABPV-R | GGAGTTTCCACATCATGAAAGG |  |  |
|  |  | Probe | FAM- CTCTGAAGAAAACTCAGTTGAAACGGAAC- BHQ-1 |  |  |
| 5 | KBV | KBV-F | ACCAGGAAGTATTCCCATGGTAAG | 79 |  |
|  |  | KBV-R | TGGAGCTATGGTTCCGTTCAG |  |  |
|  |  | Probe | HEX- CCGCAGATAACTTAGGACCAGATCAATCACA- BHQ-1 |  |  |
| 6 | IAPV | IAPV-F | TGCCCTATTTAGGGTGAGGAG | 245 |  |
|  |  | IAPV-R | GGAGTTTCCACATCATGAAAGG |  |  |
|  |  | Probe | ROX- ACTAGTGAGAACTCGGTTGAGACCCAAG- BHQ-2 |  |  |
| 7 | CBPV | CBPV-F | CGCAAGTACGCCTTGATAAAGAAC | 101 |  |
|  |  | CBPV-R | ACTACTAGAAACTCGTCGCTTCG |  |  |
|  |  | Probe | Cy5- TCAAGAACGAGACCACCGCCAGTTC- BHQ-3 |  |  |
| 8 | AmFV | AmF-For | CACGAAAGCCCTCAAATCGT | 155 | This study |
|  |  | AmF-Rev | GCGGGTAAATCCACTAAGGC |  |  |
| 9 | Lake Sinai virus | LSV1-For | GGCCGTCATGGTGGCGAATAAAATCGTCGCTGTTGTCCTTG | 198 | ^2^ |
|  |  | LSV1-R | CAACCTCTCGTAAGCAAGCC |  |  |
|  |  | LSV2-RT-F | GGTTTAGCGCTTCGCAGTTA | 333 | This study |
|  |  | LSV2-RT-R | ATTACGCACCTTCGTTTGGG |  |  |
|  |  | LSV3-RT-F | TCCCAAGAGAACCACTCACC | 273 |  |
|  |  | LSV3-RT-R | CGATTCGACGAACGATGAGG |  |  |
|  |  | LSV4-RT-F | GGGTATCTCGGTACCCACTG | 357 |  |
|  |  | LSV4-RT-R | TGCGATATGACCTCAGCACT |  |  |
| 10 | VDV | VDV-1 F | GCCCTGTTCAAGAACATG | 413 | ^3^ |
|  |  | VDV-1 R | CTTTTCTAATTCAACTTCACC |  |  |
| 11 | DWV-VDV-1 | VDV-1DWV-F | CATGGAAATGGGATCAAAC | 206 | ^4^ |
|  |  | VDV-1DWV-R | AAACGGGCTGAAAATCACAC |  |  |

Abbreviations: ABPV: acute bee paralysis virus; BQCV: black queen cell virus; CBPV: chronic bee paralysis virus; DWV: deformed wing virus; IAPV: Israeli acute paralysis virus; KBV: Kashmir bee virus; SBV: sacbrood virus; VDV: *Varroa destructor* virus; AMFV: *Apis mellifera* filamentous virus; DWV-VDV-1: deformed wing virus- *Varroa destructor* virus

**Table S3. Primers and probe sequences used to detect brood diseases and parasites**

| No. | Target | Primer | Sequence (5ꞌ→3ꞌ) | Amplicon size (bp) | Reference |
| --- | --- | --- | --- | --- | --- |
| 1 | *Paenibacillus larvae*  (AFB) | AFB-F | AAATCATCATGCCCCTTATG | 158 | ^1^ |
|  |  | AFB-R | CGATTACTAGCAATTCCGACT |  |  |
|  |  | Probe | FAM-CGTACTACAATGGCCGGTACAACG–BHQ-1 |  |  |
| 2 | *Melissococcus plutonius* (EFB) | EFB-F | TGTTGTTAGAGAAGAATAGGGGAA | 69 |  |
|  |  | EFB-R | CGTGGCTTTCTGGTTAGA |  |  |
|  |  | Probe | Cy5-AGAGTAACTGTTTTCCTCGTGACGGT-BHQ-3 |  |  |
| 3 | *Apocephalus borealis*  (phorid fly) | Phorid-F | CCTCTGTTCTACTTTCATTGGTTTAT | 255 |  |
|  |  | Phorid-R | GAGRGCCATAAAAGTAGCTACACC |  |  |
|  |  | Probe | JOE-GGCATTAGTATTACGACGCGAGAGGTG–BHQ-1 |  |  |
| 4 | *Acarapis woodi* mite | ACAR-F | CAGTAGGGCTAGATATCGATACCCGAGCTT | 247 |  |
|  |  | ACAR-R | TGAGCTACAACATAATATCTGTCATGAAGA |  |  |
|  |  | Probe | TexasRed-CAATCCACCTACAGAAAATAAAAATAAAAATCC–BHQ-2 |  |  |
| 5 | *Nosema cerana* | Nosema-F | CGGATAAAAGAGTCCGTTACC | 249 |  |
|  |  | Nosema-R | GAGCAGGGTTCTAGGGAT |  |  |
|  |  | Probe | FAM-CGTTACCCTTCGGGGAATCTTC–BHQ-1 |  |  |
| 6 | *Aspergillus flavus* (stonebrood) | ASP-F | GCTGCCCATCAAGCACGG | 127 |  |
|  |  | ASP-R | CCTACAGAGCGGGTGACAAAG |  |  |
|  |  | Probe | JOE-TGTGTGTTGGGTCGTCGTCCCCTCTC–BHQ-1 |  |  |
| 7 | *Ascosphaera apis* (chalkbrood) | ASCO-F | ATTGCGCCCTCTGGTATTC | 215 |  |
|  |  | ASCO-R | CCACTAGAAGTAAATGATGGTTAGA |  |  |
|  |  | Probe | TexasRed-GCTTGAGGGTTGCAATGACGCTCG BHQ-2 |  |  |
| 8 | *Typanosoma* | Try-RT-For | GGGCGGCATATCTGTTACAC | 276 | This study |
|  |  | Try-RT-Rev | AAGAGCCGACATCGAAGGAT |  |  |

Abbreviations: AFB: American foulbrood; EFB: European foulbrood.

**
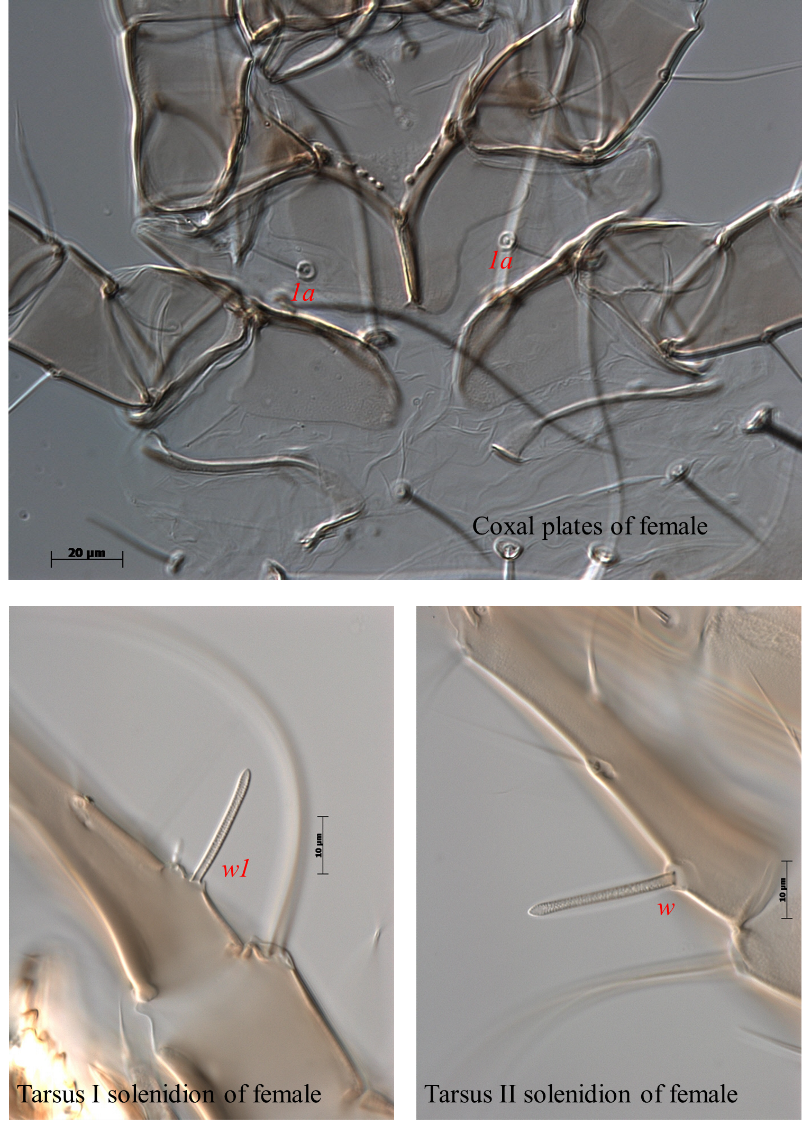
**

**Figure S1. Morphological characteristics of *Tyrophagus curvipenis*.** Coxal plate II broadly triangular, extending distally beyond apex of apodeme II; Tarsus I ω1 slender, cylindrical and slightly widened at apex, tarsus II ω slender, almost cylindrical.

**References**

1 Truong, A.-T. *et al.* Prevalence of honey bee pathogens and parasites in South Korea: A five-year surveillance study from 2017 to 2021. *Heliyon* **9**, e13494, doi:10.1016/j.heliyon.2023.e13494 (2023).

2 Runckel, C. *et al.* Temporal analysis of the honey bee microbiome reveals four novel viruses and seasonal prevalence of known viruses, *Nosema*, and *Crithidia*. *PLoS One* **6**, e20656, doi:10.1371/journal.pone.0020656 (2011).

3 Radzevičiūtė, R. *et al.* Replication of honey bee-associated RNA viruses across multiple bee species in apple orchards of Georgia, Germany and Kyrgyzstan. *J Invertebr Pathol* **146**, 14-23, doi:10.1016/j.jip.2017.04.002 (2017).

4 Zioni, N., Soroker, V. & Chejanovsky, N. Replication of *Varroa destructor* virus 1 (VDV-1) and a *Varroa destructor* virus 1–deformed wing virus recombinant (VDV-1–DWV) in the head of the honey bee. *Virology* **417**, 106–112, doi:10.1016/j.virol.2011.05.009 (2011).
